# Use of *Eisenia fetida* as a Biological Risk Marker in a Qualitative Eco Assessment Test of a Romanian Watercourse

**DOI:** 10.3390/biology11060820

**Published:** 2022-05-26

**Authors:** Romeo T. Cristina, Mihai Baroga, Eugenia Dumitrescu, Florin Muselin, Alexandru O. Doma, Dan Manea, Ioan Banatean-Dunea

**Affiliations:** 1 Departments of Pharmacology and Pharmacy, Banat University of Agriculture and Veterinary Medicine from Timisoara, Calea Aradului 119, 300645 Timisoara, Romania; baroga.mihai-dj@ansvsa.ro (M.B.); eugeniadumitrescu@usab-tm.ro (E.D.); 2 Department of Toxicology, Banat University of Agriculture and Veterinary Medicine from Timisoara, Calea Aradului 119, 300645 Timisoara, Romania; florinmuselin@usab-tm.ro (F.M.); alexandru.doma@usab-tm.ro (A.O.D.); 3 Department of Ecology, Banat University of Agriculture and Veterinary Medicine from Timisoara, Calea Aradului 119, 300645 Timisoara, Romania; dan_manea@usab-tm.ro (D.M.); ioan_banatean@usab-tm.ro (I.B.-D.)

**Keywords:** aquatic ecosystem and soil pollution, environmental monitoring, qualitative acute risk assessment, *Eisenia fetida*

## Abstract

**Simple Summary:**

In the bio-ecological perils, experts have to specify the nature of the threat. In this experiment, the aim is to have inexpensive and fast qualitative tests and the acute static tests using earthworms are of great interest due to their easy development. In Romania, 50 years of pollution of soil and rivers was repeatedly recognized, due to the great number of pollutants. Presence of pollutants and their associated sources, the soil’s revitalization and water’s revitalization was a slow process. In this regard, we tested for the first time in Southern Romania the qualitative risk of pollution with *Eisenia fetida* earthworms for two years, in 15 locations on the Jiu River, in two counties from Romania. The ISO: 11268-2:2015 acute static test was initiated, observing the ethological and bodily features of *E. fetida* earthworms for 14 days. Results revealed statistically noteworthy values (*p* < 0.05) of the riverbank margin soils which is considered polluted. The earthworms’ mortality showed a high statistical correlation related to soil samples gathered from 10 m (*p* < 0.01) and 30 m (*p* < 0.001), confirming the suspicion of deleterious chemical factors presence.

**Abstract:**

The qualitative trials were conducted by exposing earthworms to diverse contaminants sources using standard earthworms’ avoidance tests (considered useful ab initio indicators). For two years, we observed the Jiu River pollution points. We observed soil traits in 15 sampling points from two neighboring Romanian counties where Jiu River flows, by evaluating the risk of pollution on *Eisenia fetida* earthworms. The ISO: 11268-2:2015 acute static test was used, following the ethological and bodily features of *E. fetida* earthworms for 14 days, and then the results obtained for clean soils vs. those presumed polluted were statistically analyzed. Results disclosed statistically significant values (*p* < 0.05) for the two-way ANOVA and Tukey multiple comparisons tests used for the soil samples thought to be polluted. The mortality percentages by location/replica/year/county find out a high statistical correlation documenting observations related to soil samples gathered from 10 m (*p* < 0.01) and 30 m (*p* < 0.001). Compared with the control, the statistical analysis of Relative Growth Rate (RGR) (*p* < 0.05) and Specific Growth Rate (SGR) (*p* < 0.01) confirmed that, in the case of soil samples collected from 10 and 30 m from the Jiu River’s axis, the earthworms did not gain weight, qualitatively attesting the pollution suspicion/presence of chemical factors potentially pernicious for earthworms.

## 1. Introduction

The soil contamination evaluation, and its biological self-purification processes, can be identified by multiple hygienic and sanitary means. Among the various means using earthworm, qualitative avoidance tests for sublethal doses of pollutants could be practical. In this sense, *Eisenia fetida* and *E. andrei* are considered representatives of the soil fauna, in recent times, with latest available information about the ecology of earthworms and their use in diverse ecotoxicological testing, (for example: organic structures [1] and compounds [2,3], drug origin active substances [4,5] or heavy metals [4,6,7]).

Earthworm (*Eisenia fetida*) is accepted as an efficient bio-indicator for evaluation of soil pollutants [8]. *Eisenia fetida* earthworms exist in two species. They are morphologically similar, but one, *E. fetida foetida,* has a typical transverse striping on segments, whereas in the case of the variety *E. foetida andrei*, it lacks this, being identified by the spotted reddish color. Additionally, different earthworm species were used, as test organisms, such as *Aporrectodea caliginosa, Lumbricus rubellus*, and *L. terrestris,* but these are less sensitive, and the database and experience in soil testing of these species is lacking [9,10,11,12,13].

In the complex ecotoxicity studies, the elements and influences related to water and soil pollution, as an association, are assessed. As a result, more easy procedures for pollution’s early identification were designed. The amounts of detrimental, industrial, or animal wastes in ecosystems are growing logarithmically, thus each scientific study on this topic holds a great impact [14,15,16,17,18]. 

Eco-toxicological testing systems are modern methodologies applied to obtain data on the effects of soil contaminants and are often proposed to complete conventional known chemical analysis. While this testing type is employed to evaluate the soil habitat’s function, the soil testing of river locations can also be applied to obtain details about the contaminants’ presence that can reach the aqueduct through the soil retention function, new test models, utilizing earthworms being imagined, against this setting [19]. To prove the initial ecological threat’s presence, the researchers suggested these new methods, knowing that earthworms exhibit getaway behavior in response to sublethal concentrations of diverse substances [15,20,21,22,23,24,25].

Investigators confirmed the importance of using oligochaetes, especially those from the *Annelida* class, in the environment’s pollution assessment. In contrast, in other studies, the contrary was observed, for example, in the case of cypermethrin testing, when neither the concentration, nor the pollutant, or the different characteristics of the soil, triggered avoidance activities by earthworms [21]. 

On the other hand, in an evaluation, the behavior of *Eisenia fetida* on 24 insecticides was tested by Wang et al. (2012), who demonstrated that toxicity was closely related to the group of substances and the test method [22]. The results allowed the conclusion that avoidance tests have an advantage, namely the short duration (usually 48 h), are convenient, have enough sensitivity, although often indicative, is very valuable from an ecological point of view as they provide early behavioral reactions, easily observable, even at sublethal or sub-toxic concentrations of environmental contaminants. In addition, it is to be mentioned the reproducibility of these investigations [25,26].

In view of notable relationship of multi-biological responses in earthworm under stress we agreed out the hypothesis that oxidative stress, mitochondrial bioenergetics and burrowing could perform as extensive biomarker for soil pollution, and these can be easily associated with our test outcome [3]. This study is based on our cummulative experience in this type of eco-risk approach. As a novelty, this is the first study ever made in Southern Romania applying a qualitative methodology with *E. fetida* earthworms using an acute static test in association with the Relative Growth Rate (RGR) and Specific Growth Rate (SGR) indicators. Here, we monitored the significance of Jiu water pollution menace in two counties from Southern Romania, measured on neighboring soil characteristics. Upon our knowledge this is the first study made in this topic in Southern Romania.

## 2. Materials and Methods

### 2.1. Locations Analyzed

The soil samples used sediments gathered from locations along the Jiu River route in Gorj and Dolj counties, Southern Romania, where the Jiu River has been in the past the subject multiple threats related to pollution and in association with the existence of two “historical” pollutants, the Turceni and Ișalnița electro thermal power plants in the area. Here, the water and soil analysis consistently revealed the NH_3_, NO_2_, SO_2_, emission plus the heavy metals (e.g., Pb, Ni, Co, Cr, Cd, Cu) presence in the soil, and the existence of NH^4+^, iron, and NO_3_^_^ in water.

Study was performed for two successive years, in January/February, and, respectively, July/August, in 15 locations and from four points settled at: 10–30–50–100 m, from the axis of the Jiu River. To diminish any error, the samples taken from the 15 locations were compared with reference soil samples, considered polluted free (Control), taken under similar conditions, from a public park from Craiova (Dolj county), according to the instructions: Soil quality effects of pollutants on earthworms (ISO11268-2- Geneva, Switzerland, 2012, revised 2015). The uncontaminated soil was analyzed at the Craiova Office of Pedologic and Agrochemical Studies (OSPA), being considered representative: pH = 8.0; electrical conductivity (EC) = 0.15 dS × m^−1^; and a percentage of exchangeable sodium (ESP) = 0.45%), having similar properties of pH, organic carbon content and texture with the tested soils [27,28,29].

**Figure 1:**
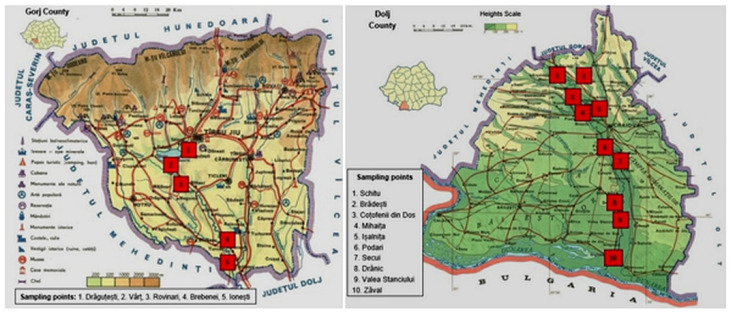
Geographical repartition of the soil units in Dolj and Gorj Counties [28,29].

The geographical distribution of the soil unit from the studied Dolj and Gorj counties it is shown in Figure 1, and Supplementary Table S1 it shows the location of soil samplings points and the main parameters of: pH, atmospheric pressure (hPa), relative humidity (%), and humidity at the sampling moment.

Soil samples were collected from a depth of 15–20 cm and adjacent to the Jiu Riverbed, choosing four extraction points, located at 10 m (samples a); 30 m (b); 50 m (c) and, respectively 100 m (samples c) distance from the river axis.

### 2.2. Testing Methodology

The ethological and somatic characteristics of the *Eisenia fetida* earthworms were followed for a 14 -day period, after which the results were evaluated for clean soil samples (considered Control) and for the soils presumed contaminated (considered test soils). The experimental technique used is presented by ISO 11268-2: 2015, for testing the toxicity of contaminated soils, using earthworms without soil regeneration (e.g., *Eisenia fetida, E. andrei*, or *Lumbricus terrestris*) [26,27,30].

In the study, for each soil sample collected from the 15 test-established locations were used five earthworms/evaluation; therefore, the experiment was reproduced for each test location/distance/replica, using 600 earthworms per year, and 20 earthworm/sample/replica/year were considered Control. Following the instructions of Fründ et al. (2009), after sampling the soil specimens (minimum 750 g) were well homogenized, comparable amounts being introduced into the testing devices, in three-liter glass jars, filling with soil being accomplished in 3 cm successive layers, packed with the fist to 1.4 g × cm^3^ force [31].

Knowing the *Eisenia fetida* choice for damp soils with a relative humidity of 70–80%, an important point was to maintain the Water Holding Capacity (WHC).

Following the instructions of Fründ et al. (2009), this was completed by wetting at daily intervals and covering with perforated foils to prevent the soil’s moisture loss, and avoiding stressing the oligochaetes. During the testing, the temperature was maintained, in the range of 20 ± 2 °C, and the photoperiod light-dark ratio was 16:8 h/day [20,31,32]. After balancing the water retention, the earthworms were deposited in the test devices and oatmeal and mashed potatoes were given as food [26,27].

The mortality assessment was visually performed: at one hour after introduction into the test pots (observing their presence/absence on the soil’s surface); and after seven days (when mortality was evaluated). The final examination was made on day 14 (with the mortality/behavioral changes registration).

The living earthworms were counted, per each experimental replica, considering them dead, when they no longer reacted to the gentle mechanical stimuli applied to the worms’ cephalic extremity. The earthworms have been considered lifeless when they were not found in the observed soil samples on days 7 and 14. According to ISO 11268-1:2015 [27], the effect on earthworms’ survival during the 14 testing days, was defined as mortality rate and calculated according to the formula:

Mortality (%) = E_0_ − E_t_/E_0_ × 100 (1)

where: E_0_ is the number of earthworms at the beginning of the exposure and E_t_ is the number of live worms at the end of the exposure period.

Earthworms were weighed at the beginning and the end of the examination and according to Sogbesan and Ugwumba, the earthworm growth rate was represented mathematically as Relative Growth Rate (RGR) and Specific Growth Rate (SGR) [33].

The RGR is the percentage ratio of weight gained to initial body weight as:

RGR = Weight R gain/Initial body weight × 100(2)

SGR was calculated as a percentage weight gained over time using the formula:

SGR = Log w_f_ − Log w_i_/t × 100 (3)

where: log w_f_ (logarithm of the final weight), log w_i_ (logarithm o the initial weight) and t, is the experiment time.

### 2.3. The Statistic Analysis

The evaluation of the difference between groups, was ascertained using the two-way ANOVA, associated with Tukey’s multiple comparison test using Graph Pad Prism 6.0 program for Windows (Graph Pad Software, San Diego, CA, USA). Statistical values were expressed as ±SEM (Standard Error of the Mean): where *, means 0.01 ≤ *p* < 0.05, significant; **, means 0.001 ≤ *p* < 0.01, very significant; ***, means *p* < 0.001, extremely significant.

## 3. Results

**Figure 2:**
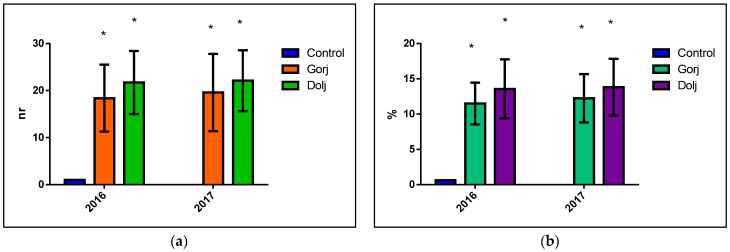
The statistical analysis of the results of the worm counts, compared with the Control. The (**a**) image represent the number of dead earthworms, and (**b**) image represent the percentage of dead earthworms, where * means, *p* < 0.05).

The results of the earthworms’ counters have compared with the Control, the results indicating statistically significant values for the two-way ANOVA and for Tukey’s multiple comparisons test for the soils tested, and for both visited counties (*p* < 0.05) (Figure 2).

Analyzing per each year and county, the mortality in earthworms registered their most elevated value in 2016, in Gorj county, in the sampling points (P): P5 (37/23.125%); P4 (36/22.50%), and P3 (17/16.25%). The values for 2017 confirmed the previous year’s mortality tendency, respectively: P5 (36/22.50%); P4 (36/22.50%), even an increase, in the case of P3 (26/16.25). In the case of Dolj county, in 2016, the top mortality was detected in the case of: P8 (55/34.375%); P7 (50/31.25%); P6 (43/26.875%) and, respectively, P5 (32/20.0%). In year 2017, the highest values of the mortality were, in descending order at: P8 (50/31.25%); P7 (50/31.25); P6 (45/28.125%) and P5 (35/21.875%).

Regarding the distances from where the samples were gathered, the highest mortality also statistically confirmed appeared from 30 m (*p* < 0.001), and 10 m (*p* < 0.01). At the 50 m from the Jiu axis, the mortality dropped significantly: in Gorj between 0 and 9/5.625%, and, respectively, 0 and 8/5.0% dead earthworms in Dolj County. Only in one case, mortality at 100 m it was recorded (1/0.625%).

**Figure 3:**
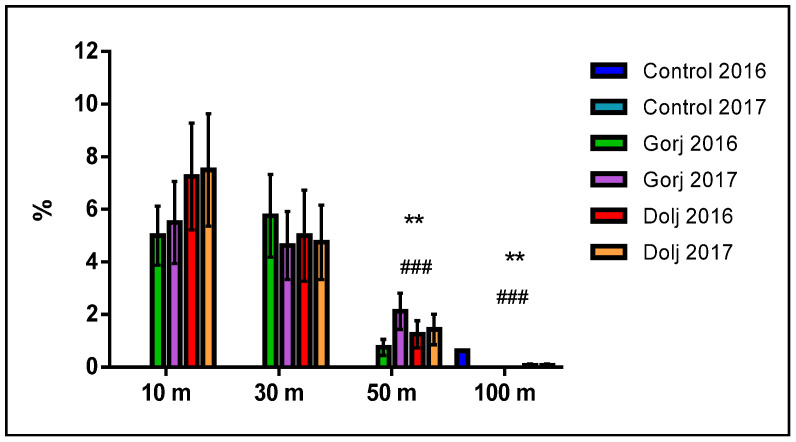
Statistical comparison by county and by year and the statistical significance of soils pollution sampled from 10, 30, 50 and 100 m vs. the earthworm mortality rate. (Compared with 10 m, where **, means = *p* < 0.01 and compared with 30 m, where ###, means *p* < 0.001).

Figure 3 presents the statistical comparison per each county and year and the importance of the biological impact of soils harvested from 10, 30, 50, and 100 m on the mortality rate of earthworms.

During this study, no behavioral modifications of earthworms were observed. Consequently, to investigate if the earthworm mortality has a direct correlation with potentially lethal exogenous polluting or chemical factors for the living organisms, we completed this study also with the earthworms’ Relative Growth Rate and Specific Growth Rate evaluation [33].

**Figure 4:**
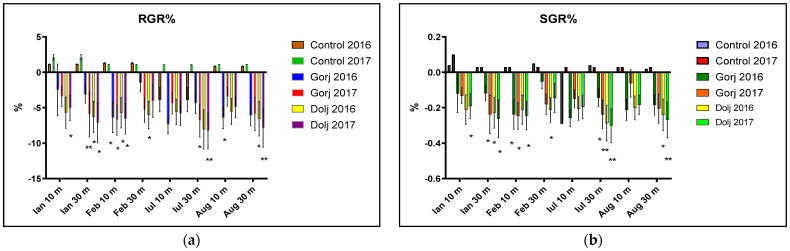
Statistical analysis of the Relative Growth Rate (RGR) (**a**) and the Specific Growth Rate (SGR) (**b**) per years/replicas/county, compared with Control unpolluted soils, which confirms the potential polluting effect of soils gathered at 10 and 30 m from the Jiu River’s axis. (Where *, means *p* < 0.05, and **, means *p* < 0.01).

The effect on earthworm growth rate was expressed mathematically as a calculation formula for RGR (as the percentage ratio between the weight gained and the initial weight) and SGR (calculated as weight gained percentage in time). Compared with the Control, the statistical analysis of RGR and SGR proved that in the case of soil samples gathered from 10 and, respectively, 30 m from the Jiu River’s axis, the earthworms did not gain weight, confirming the qualitative fact of polluting or the existence of chemical factors potentially lethal (to these organisms), the results being statistically significant for both the RGR and SGR parameters (* *p* < 0.05, ** *p* < 0.01), as shown in Figure 4.

## 4. Discussion

We consider that the avoidance tests for polluted soils are helpful for the practice of the eco-toxicological assessment due to the multiple benefits they offer: they can be efficiently completed, are affordable, have a short duration, and the results can be certainly used to initially evaluate contamination from areas with unknown eco-toxicological conditions. Acknowledged avoidance studies have shown that, *Eisenia fetida* species of oligochaetes are very suitable for eco-toxicity studies, this species having the characteristic of a shallow digging (only 10–20 cm) and preference rich organic soils. This is why we selected this species for qualitative eco-toxicity analysis, as other authors from this area [31,32,34,35,36,37].

One of the first detailed studies to test the effect of various harmful chemicals on earthworms was conducted by Edwards and Bohlen in 1992, and they revealed for the first time the need to standardize methodology for assessing environmental pollution [29].

Recent knowledge revealed that soil pollutants could induce manifold biological responses translated as integrated biomarker response (IBR) in earthworms, the most important ones being: the oxidative stress [38], molecular damages [39], correlated transcriptional regulation, and, respectively, the individual-level responses [3,19,40]. In their comprehensive studies, Li et al. (2019) sorted out the biomarkers and then associated the obsrved alterations median scores, in agreement with their outcomes [1,4,6].

In the previous decade, integrated biomarker response indexes (IBR) imagined are including various biological sources associated with many general alterations found in earthworms with pollution. In all cases, the phenotypic and corporal answers were almost identical: the earthworm death with associated behavior and body modifications.

To extend more these biomarkers, Zhao et al. (2020) standardized the statistical deviation of each biomarker and then constructed the incorporated biomarker response version 2 (IBRv2), to reflect more accurately the pollutants’ activity. In this respect, the advantage of present qualitative study is that, even though, the exact nature of the pollutant structure is not known, them presence is swiftly mirrored in the behavior of *Eisenia fetida*, with convincible results, and firmly related to the area’s pollution determining as other authors observed [41].

Both these strategies using the earthworms as bio-indicators, may deliver important views for the environmental eco-toxicity assessment and could be additionally explored. The interactions between pollution biomarkers and earthworms, evaluated with an easy qualitative methodology could be of interest for the initial pollution suspicion studies.

Yeardley et al. (1996) established that *Eisenia andrei* and *E. fetida* species can avoid sublethal concentrations of chemical pollutants in soils after short exposures, for one to two days [32]. In *Eisenia fetida*, species, Yeardley (1996) noted definite avoidance responses at 48 h after test began [27], bu also other exposure periods, ranging from 24 to 72 h, presented in the main stream, confirmed the avoiding behavior of the earthworms contaminated with organic pollutants at concentrations similar to those that had a visible impact [10,20,35].

Our observations are consistent with those of Nadal da Luz et al. (2004), who confirmed that the earthworm population density declines with the pollutant concentration increase [35]. As well, studies of Feisthauer (2003), established that one great benefit of avoidance tests, even if they are only qualitative is that they are fast and low-cost alternatives, to the extended “breeding tests”. These simple tests can set the background for the initial screening of potentially contaminated soils [36].

In this aim, our research also confirmed that in *E. fetida,* avoidance behavior is a factor entirely measurable as a statistical value, confirming other studies which have shown that the behavioral parameter is similar or even more sensitive than other parameters (e.g., cell proliferation or somatic growth) [37].

In our opinion, if completed accurately, this static test using *Eisenia fetida* worms has high specificity, generating qualitative outcomes and could be used to distinguish the presence of an eco-toxicological threat, providing valuable initial data on the pollution development and extent. In this regard, the statistical analysis we used, the bidirectional ANOVA associated with Tukey’s multiple comparison test, evaluating the differences between groups we regarded as significant, when the differences had the value of *p* < 0.05 or less. Values expressed as standard error of the mean provided accuracy, being a practical alternative for this type of testing, recommended also by other authors [42].

The results showed that out of 1200 soil samples tested from the 15 different locations, high earthworm mortality rates were recorded (*p* < 0.05 or less), confirming the potential ecological risk in seven collection locations: three in Gorj County and four in Dolj County. Additionally, the added statistical analysis of the RGR and SGR parameters/years/replica/counties, compared with the Control, confirmed the potential polluting effect of soils sampled at 10 and 30 m from the Jiu axis (*p* < 0.05, and *p* < 0.01).

## 5. Conclusions

The used 14-day acute static test can be performed with significant results in testing environmental ecotoxicity. The results interpretation can provide the basis for statistically significant assessments that can be compared with other results in the field.

The statistic evaluation of the soil samples compared with the Control for RGR/SGR parameters, statistically confirmed the values for the 10 m (*p* < 0.05), and 30 m (*p* < 0.01) sampling distances, and proven a high statistical connection with a pollution source. In our opinion, this is a simple, cheap and quick assessment that should be included in a set of tests for the early qualitative screening, an orientative tool in soil contamination assessing, indicating the soil’s potential bio-risk.

## Data Availability

All data are included in this manuscript.

## Supplementary Materials

The following supporting information can be downloaded at: https://www.mdpi.com/article/10.3390/biology11060820/s1, Table S1: Soil sampling locations from Jiu’s riverbanks in Gorj and Dolj Counties and the main parameters.
