# Supplementary material for: Use of Eisenia fetida as a Biological Risk Marker in a Qualitative Eco Assessment Test of a Romanian Watercourse
Source: Biology (Basel). 2022 May 26;11(6):820. doi: 10.3390/biology11060820 (PMC9219812; doi:10.3390/biology11060820)
Supplement: Supplementary file 1 [file biology-11-00820-s001.zip › biology-1743729-supplementary.pdf]

Supplementary Table S1. Soil sampling locations from Jiu's riverbanks in Gorj and Dolj Counties and the main parameters

Sample no.	GPS Lat.	GPS Long.	Parameter	2016 January	2016 February	2016 July	2016 August	2017 January	2017 February	2017 July	2017 August
**Gorj County**											
P1 (upstream) **Drăguțești**	N 44°58'02.8 "	E 23°12'54.1"	Sampling time	10.00	9.45	9.45	10.00	9.45	9.45	10.00	10.00
			Atmospheric pressure (hPa)	1019	1015	1015	1020	1018	1013	1015	1020
			Relative humidity (%)	63	61	59	62	55	63	60	60
			pH	7.0	7.1	7.1	7.2	7.0	7.2	7.2	7.1
			Moisture at sampling	dry	dry	dry	dry	cloudy	cloudy	dry	dry
P2 (upstream) **Vîrț**	N 44°56'55.0"	E 23°07'57.3"	Sampling time	11.00	10.45	11.15	11.00	10.45	11.00	11.15	11.00
			Atmospheric pressure (hPa)	1018	1012	1015	1019	1015	1015	1020	1018
			Relative humidity (%)	59	60	64	65	58	59	60	63
			pH	7.1	7.3	7.2	7.2	7.3	7.4	7.4	7.5
			Moisture at sampling	dry	cloudy	dry	cloudy	dry	dry	dry	dry
P3 (downstream) **Rovinari**	N 44°54'11.6"	E 26°09'29.1"	Sampling time	12.00	11.30	11.45	11.45	11.45	11.45	12.00	12.00
			Atmospheric pressure (hPa)	1018	1012	1015	1020	1014	1015	1019	1017
			Relative humidity (%)	59	60	64	65	58	59	60	63
			pH	7.3	7.2	7.5	7.3	7.4	7.6	7.4	7.4
			Moisture at sampling	cloudy	cloudy	dry	dry	dry	dry	dry	dry
**P4** (downstream) Brebenei	N 44°38'37.6"	E 23°26'21.8"	Sampling time	13.00	12.30	12.30	12.30	12.45	12.45	12.45	12.45
			Atmospheric pressure (hPa)	1020	1019	1019	1015	1015	1016	1015	1018
			Relative humidity (%)	55	58	61	63	57	56	63	62
			pH	7.3	7.2	7.4.	7.4	7.4	7.2	7.5	7.3
			Moisture at sampling	dry	cloudy	dry	cloudy	dry	dry	dry	dry
**P5** (downstream) Ionești	N 44°37'11.00"	E 23°27'1.12"	Sampling time	14.00	13.45	13.30	13.45	13.45	13.30	13.45	13.30
			Atmospheric pressure (hPa)	1018	1012	1015	1020	1018	1017	1015	1015
			Relative humidity (%)	54	54	61	62	57	55	62	63
			pH	7.6	7.5	7.8	7.4	7.7.	7.7.	7.4	7.5
			Moisture at sampling	dry	dry	dry	dry	dry	dry	dry	dry
**Dolj County**											
**P1 (upstream) Schitu**	N 44°30'44.50"	E 23°30'38.93"	Sampling time	9.00	9.00	9.15	9.00	9.15	9.00	9.00	9.15
			Atmospheric pressure (hPa)	1018	1012	1015	1020	1019	1015	1015	1020
			Relative humidity (%)	55	54	60	63	57	55	62	61
			pH	7.6	7.4	7.7	7.6	7.6	7.8	7.6	7.8
			Moisture at sampling	cloudy	cloudy	dry	dry	dry	cloudy	dry	dry
**P2 (upstream) Brădești**	N 44°29'27.86"	E 23°35'52.27"	Sampling time	10.00	10.00	10.15	10.00	10.00	10.00	10.15	10.15
			Atmospheric pressure (hPa)	1019	1016	1016	1014	1015	1020	1019	1016
			Relative humidity (%)	55	55	64	61	53	56	60	64
			pH	7.5	7.5	7.6	7.4	7.5	7.7	7.7	7.6
			Moisture at sampling	dry	dry	cloudy	dry	dry	cloudy	dry	dry
**P3 (upstream) Coțofenii din Dos**	N 44°24'40.11"	E 23°40'57.86"	Sampling time	11.00	11.00	11.00	11.15	11.15	11.00	11.15	11.15
			Atmospheric pressure (hPa)	1015	1020	1019	1016	1018	1012	1015	1020
			Relative humidity (%)	55	52	63	65	50	53	63	64
			pH	7.5	7.4	7.5	7.6	7.6	7.5	7.4	7.6
			Moisture at sampling	dry	dry	cloudy	cloudy	dry	dry	cloudy	cloudy

**P4 (upstream) Mihăița**	N 44°21'58.85"	E 23°42'34.97"	Sampling time	12.00	12.00	12.15	12.00	12.00	12.15	12.15	12.00
			Atmospheric pressure (hPa)	1014	1018	1012	1015	1015	1017	1018	1018
			Relative humidity (%)	55	52	63	61	56	55	64	64
			pH	7.7	7.7	7.8	7.6	7.6	7.8	7.6	7.8
			Moisture at sampling	cloudy	dry	dry	dry	cloudy	cloudy	cloudy	cloudy
**P5 (downstream) Ișalnița**	N 44°15'38.40"	E 23°47'6.23"	Sampling time	13.00	13.15	13.00	13.00	13.15	13.15	13.00	13.15
			Atmospheric pressure (hPa)	1018	1016	1016	1013	1017	1016	1018	1016
			Relative humidity (%)	52	53	65	62	54	55	63	63
			pH	7.3	7.2	7.1	7.1	7.2	7.2	7.0	7.2
			Moisture at sampling	dry	dry	cloudy	dry	cloudy	cloudy	cloudy	cloudy
**P6 (downstream) Podari**	N 44°11'8.62"	E 23°50'55.37"	Sampling time	14.00	14.00	14.00	14.15	14.00	14.00	14.00	14.15
			Atmospheric pressure (hPa)	1019	1016	1018	1016	1020	1019	1016	1019
			Relative humidity (%)	50	51	62	64	50	50	62	64
			pH	7.2	7.2	7.0	7.2	7.1	7.1	7.0	7.1
			Moisture at sampling	dry	dry	dry	dry	dry	dry	dry	cloudy
**P7 (downstream) Secui**	N 44° 1'21.82"	E 23°52'41.83"	Sampling time	15.15	15.00	15.00	15.00	15.15	15.15	15.00	15.00
			Atmospheric pressure (hPa)	1016	1015	1019	1016	1018	1016	1018	1016
			Relative humidity (%)	50	50	63	62	51	53	63	63
			pH	7.3	7.2	7.1	7.3	7.2	7.1	7.2	7.3
			Moisture at sampling	dry	dry	dry	dry	dry	dry	cloudy	dry
**P8 (downstream) Drănic**	N 43°49'3.45"	E 23°49'36.68"	Sampling time	16.00	16.00	16.15	16.15	16.00	16.00	16.15	16.15
			Atmospheric pressure (hPa)	1016	1015	1019	1016	1016	1018	1016	1015
			Relative humidity (%)	51	53	62	65	50	55	64	64
			pH	7.5	7.7	7.5	7.6	7.5	7.7	7.5	7.5
			Moisture at sampling	dry	cloudy	dry	cloudy	dry	cloudy	cloudy	cloudy
**P9 (downstream) Valea Stanciului**	N 43°58'42.3"	E 23°52'43.2"	Sampling time	17.00	17.00	17.50	17.00	17.15	17.00	17.15	17.00
			Atmospheric pressure (hPa)	1020	1019	1016	1019	1016	1018	1016	1015
			Relative humidity (%)	53	52	61	63	53	53	63	61
			pH	7.8	7.6.	7.7.	7.6	7.7	7.7	7.6	7.6
			Moisture at sampling	dry	dry	dry	dry	dry	cloudy	dry	dry
**P10 (downstream) Zăval**	N 43o85'12.19"	E 23o84'83.37"	Sampling time	18.00	18.15	18.00	18.15	18.15	18.00	18.15	18.00
			Atmospheric pressure (hPa)	1019	1016	1018	1016	1015	1020	1019	1016
			Relative humidity (%)	51	51	64	62	53	55	62	63
			pH	7.9	7.8	8.0	7.9	7.9	8.0	7.9	7.9
			Moisture at sampling	dry	dry	cloudy	dry	dry	cloudy	dry	cloudy
**Control**											
**Craiova**	N 44°30'11.6"	E 23°80'69.9"	Sampling time	20.00	20.00	20.15	20.00	20.15	20.00	20.15	20.15
			Atmospheric pressure (hPa)	1018	1016	1015	1020	1019	1016	1019	1016
			Relative humidity (%)	50	51	63	63	53	55	63	64
			pH	7.8	7.7	7.9	7.7	7.7	7.9	7.8	7.9
			Moisture at sampling	dry	dry	dry	dry	dry	cloudy	dry	cloudy
